# Triglyceride Glucose-Waist Circumference Is Superior to the Homeostasis Model Assessment of Insulin Resistance in Identifying Nonalcoholic Fatty Liver Disease in Healthy Subjects

**DOI:** 10.3390/jcm11010041

**Published:** 2021-12-23

**Authors:** Hwi Seung Kim, Yun Kyung Cho, Eun Hee Kim, Min Jung Lee, Chang Hee Jung, Joong-Yeol Park, Hong-Kyu Kim, Woo Je Lee

**Affiliations:** 1 Department of Internal Medicine, Asan Medical Center, University of Ulsan College of Medicine, Seoul 05505, Korea; jennyhsk212@gmail.com (H.S.K.); chjung0204@gmail.com (C.H.J.); jypark@amc.seoul.kr (J.-Y.P.); 2 Asan Diabetes Center, Asan Medical Center, Seoul 05505, Korea; 3 Department of Internal Medicine, Hallym University Sacred Heart Hospital, Hallym University College of Medicine, Anyang 14068, Korea; yukycyk@gmail.com; 4 Department of Health Screening and Promotion Center, Asan Medical Center, University of Ulsan College of Medicine, Seoul 05505, Korea; greenk27@hanmail.net (E.H.K.); neige0126@naver.com (M.J.L.)

**Keywords:** insulin resistance, non-alcoholic fatty liver disease, obesity, triglyceride-glucose index

## Abstract

The triglyceride glucose (TyG) index has been suggested as a marker for insulin resistance; however, few studies have investigated the clinical implications of markers that combine obesity markers with the TyG index. This study aimed to investigate the associations between non-alcoholic fatty liver disease (NAFLD) and TyG-related markers in healthy subjects in Korea. We enrolled 21,001 asymptomatic participants who underwent hepatic ultrasonography. The homeostasis model assessment of insulin resistance (HOMA-IR), TyG index, TyG-body mass index, and TyG-waist circumference (WC) were subsequently analyzed. NAFLD was diagnosed using hepatic ultrasonography. A multiple logistic regression analysis was performed to evaluate the associations between the quartiles of each parameter and the risk of NAFLD. The increase in the NAFLD risk was most evident when the TyG-WC quartiles were applied; the multivariate-adjusted odds ratios for NAFLD were 4.72 (3.65–6.10), 13.28 (10.23–17.24), and 41.57 (31.66–54.59) in the 2nd, 3rd, and 4th TyG-WC quartiles, respectively, when compared with the lowest quartile. The predictability of the TyG-WC for NAFLD was better than that of the HOMA-IR using the area under the curve. The TyG-WC index was superior to the HOMA-IR for identifying NAFLD in healthy Korean adults, especially in the non-obese population.

## 1. Introduction

Insulin resistance (IR) is characterized by an insufficient physiological response to the effects of insulin, resulting in compensatory hyperinsulinemia [1,2]. IR is a major contributor to the development of type 2 diabetes and has been found to be associated with numerous other metabolic diseases, such as metabolic syndrome and dyslipidemia [3]. IR is also a key contributor to the pathophysiology of nonalcoholic fatty liver disease (NAFLD), which has recently been recognized as a hepatic component of metabolic syndrome [4]. Furthermore, it has recently suggested that NAFLD is a systemic disease which plays a critical role in metabolic syndrome; accordingly, the concept metabolic-dysfunction-associated fatty liver disease (MAFLD) has been proposed [5]. Excessive visceral adiposity in MAFLD, leading to a pro-inflammatory state, is an important risk factor for obesity-related complications such as colonic diverticulosis, which has the bi-directional relationship between metabolic syndrome or NAFLD [6]. NAFLD is a common liver disease characterized by fat accumulation in the liver in those individuals who do not drink heavily (>210 g in males and >150 g in females per week) [7,8]. While simple steatosis is regarded as benign [7,9], a subset of NAFLD patients develop nonalcoholic steatohepatitis (NASH), which is a more unfavorable condition, as it may progress to fibrosis and cirrhosis, both of which have serious clinical consequences [10]. NASH is a chronic and progressive disease, which can predispose patients to develop liver cirrhosis and hepatocellular carcinoma (HCC) [11]. The deleterious effects of NAFLD are not limited to the liver; NAFLD can damage multiple organs via systemic low-grade inflammation. Recently, it has become clear that patients suffering from NAFLD might be at higher risk of developing various infections, including urinary tract infection, pneumonia, *Helicobacter pylori*, coronavirus disease 2019, and *Clostridioides difficile* [12].

The hyperinsulinemic-euglycemic clamp (HEC) technique, which was suggested by DeFronzo, is generally recognized as the gold standard for quantifying IR [13]. However, it is impossible to employ in a real-world clinical practice setting due to the inconvenience and expense associated with the technique [14]. Instead, the homeostasis model assessment of insulin resistance (HOMA-IR) is widely used to measure IR in both clinical practice and research [15]. The triglyceride glucose (TyG) index, which is a combination of the triglyceride (TG) and fasting plasma glucose (FPG) levels, has recently been shown to have a high sensitivity and specificity for identifying IR [14,16,17,18]. This index has the benefit of being derived from the TG and FPG levels, both of which have been verified for their role in IR and are widely used in clinical practice [14,19,20,21]. Furthermore, several studies have shown that TyG-related indices that integrate both obesity markers (i.e., body mass index [BMI] or waist circumference [WC]) and the TyG index for IR are more successful than the TyG index alone [22,23,24]. However, to the best of our knowledge, there is limited evidence that these markers are effective for diagnosing individuals with NAFLD.

Therefore, in this study, we sought to investigate the use of various TyG-related indices (TyG index, TyG-BMI, and TyG-WC) for identifying NAFLD in a healthy Korean population and to compare the usefulness of these indices with the HOMA-IR, which is the classical marker for IR.

## 2. Materials and Methods

### 2.1. Study Population

**Figure 1:**
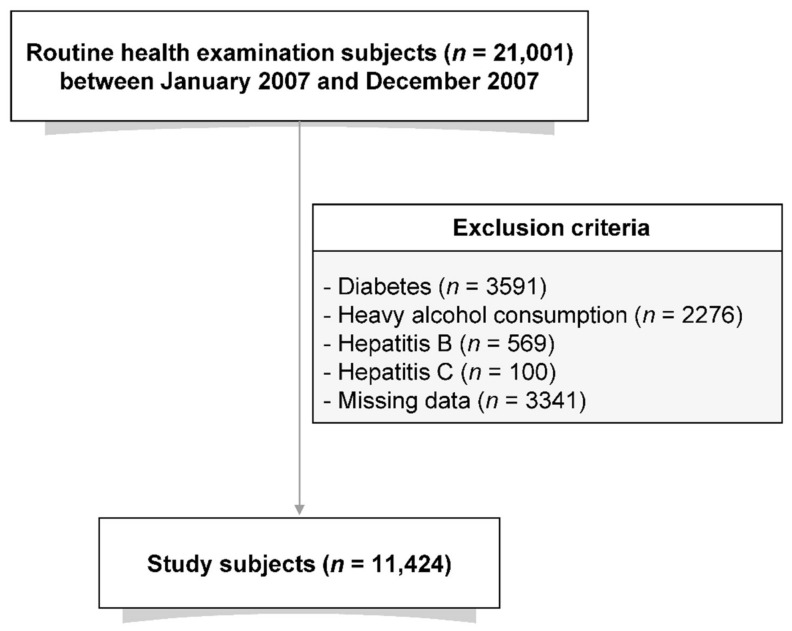
Flow diagram showing the selection process of the study population.

Individuals who underwent hepatic ultrasonography at the Asan Medical Center (Seoul, Korea) between January 2007 and December 2007 were enrolled in this study. Each participant completed a self-reported questionnaire that included their medical and surgical histories, prescription medications, and alcohol use. Participants were classified based on alcohol use as non-drinker (alcohol consumption ≤one time/week) or drinker (two or three times/week). The study cohort was initially comprised of 21,001 individuals. Participants with diabetes (*n* = 3591) or patients on lipid-lowering medications (*n* = 1411) were excluded. Participants with excessive alcohol consumption (≥four times/week) were subsequently excluded, as were those who tested positive for hepatitis B (*n* = 569) and/or hepatitis C virus (*n* = 100). Subjects with missing laboratory data (*n* = 3341) were also excluded. After the ineligible participants were excluded, the final study population consisted of 10,585 individuals (Figure 1).

### 2.2. Clinical and Laboratory Measurement

The following data were gathered for each of the participants: height, weight, systolic blood pressure (BP), diastolic BP, hemoglobin A1c, fasting glucose, lipid parameters, liver enzymes, and high-sensitivity C-reactive protein level. Subjects were to stand with their feet apart by 25 to 30 cm to measure the WC at about 3 cm above the point of the anterior superior iliac spine. All clinical and laboratory measurements were obtained at the same time. Insulin resistance was assessed using the HOMA-IR with the following formula: fasting insulin [µIU/mL] × fasting glucose [mg/dL])/405 [25]. The other TyG-related parameters were calculated using the following formulae: TyG index = Ln [TG (mg/dL) × FPG (mg/dL)/2]; TyG-BMI = TyG index × BMI (kg/m^2^); and TyG-WC = TyG index × WC (cm) [17,26].

### 2.3. Definition of NAFLD

NAFLD was diagnosed using hepatic ultrasonography by an experienced radiologist blinded to the patients’ health data and was defined as a diffuse increase in the echogenicity of the liver compared to that of the kidneys [27]. US was done on the same day as the clinical and laboratory measurements.

### 2.4. Statistical Analysis

The continuous variables that followed a normal distribution are expressed as the mean ± standard deviation (SD), and those that were not normally distributed are expressed as the median (and interquartile range). The Student’s *t* test, Mann–Whitney U test, and chi-square test were employed, as appropriate, to compare the demographic and biochemical features between the NAFLD subgroups, and a two-tailed *p* value < 0.05 was considered statistically significant. The odds ratios (ORs) and 95% confidence intervals (CIs) for the NAFLD subgroups were assessed using a logistic regression analysis. We constructed receiver operating characteristic (ROC) curves, calculated areas under the curve (AUC), and compared the AUCs using the DeLong method [24] to assess the value of the NAFLD detection parameters. MedCalc^®^ version 11.12.0 for Windows (MedCalc Software, Mariakerke, Belgium) was used to calculate the AUCs. All the statistical analyses, except the ROC curve analysis, were performed using SPSS, version 21 (IBM Corp., Armonk, NY, USA). *p* values < 0.05 were considered statistically significant.

## 3. Results

### 3.1. Clinical and Biochemical Characteristics of the Study Participants

The baseline biochemical and clinical characteristics of the study subjects according to the presence of NAFLD are shown in Table 1. Among the 11,124 participants, the prevalence of NAFLD was 31.9% (*n* = 3554). The study population had a mean (±SD) age of 48.1 ± 8.7 years and a mean BMI of 23.6 ± 2.8 kg/m^2^. Compared with the non-NAFLD individuals, the patients with NAFLD were more likely to be older and have a worse overall metabolic profile, which included the BMI, BP, FPG, uric acid, and serum lipid profiles (all *p* < 0.001). Notably, the HOMA-IR and TyG-related indices were all significantly higher for the patients with NAFLD than for those without the disease (all *p* < 0.001).

### 3.2. Relationships between NAFLD and the HOMA-IR and TyG-Related Markers

**Figure 2:**
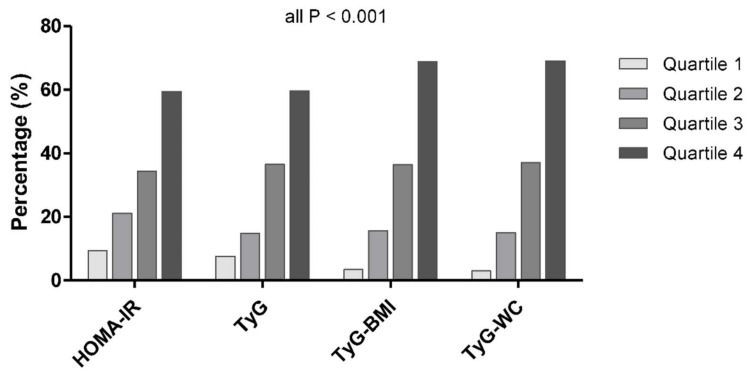
Proportion of the participants with NAFLD according to the HOMA-IR, TyG, TyG-BMI, and TyG-WC quartiles.

When categorizing the metabolic parameters into quartiles, we observed a dose–response association between all the parameters and NAFLD (all *p* < 0.001 for the linear trend) (Figure 2 and Table S1). There was a marked positive association between NAFLD and both the TyG-BMI and TyG-WC; the prevalence of NAFLD increased from 3.4% to 15.7% to 36.4% to 68.8% across the increasing TyG-BMI quartiles, and 3.0% to 15.1% to 37.0% to 69.0% across the TyG-WC quartiles (all *p* for trend <0.001).

**Figure 3:**
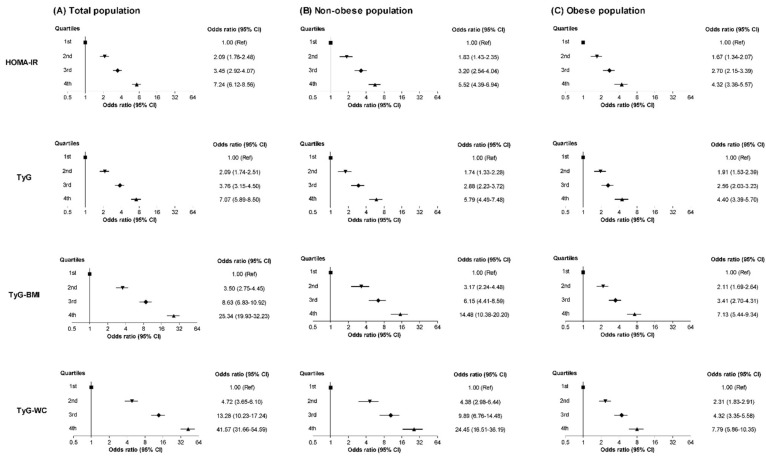
The NAFLD ORs (95% CI) according to the quartiles of HOMA-IR, TyG, TyG-BMI, and TyG-WC in the (**A**) total population, (**B**) non-obese population, and (**C**) obese population.

The NAFLD ORs were calculated according to the metabolic parameter quartiles (Figure 3 and Table S2). In general, the NAFLD ORs increased in the 2nd, 3rd, and 4th quartiles compared to the respective 1st quartile of the metabolic parameters. The increase in the risk according to the higher quartiles was most pronounced when the TyG-WC was applied; even after a full adjustment, the NAFLD ORs and 95% CIs were 4.72 (3.65–6.10), 13.28 (10.23–17.24), and 41.57 (31.66–54.59) for the subjects in the 2nd, 3rd, and 4th quartiles of the TyG-WC, respectively, compared with those in the 1st quartile. The multivariable-adjusted ORs (95% CIs) for the 4th quartiles of the HOMA-IR, TyG, and TyG-BMI were 7.24 (6.12–8.56), 7.07 (5.89–8.50), and 25.34 (19.93–32.23), respectively, compared to the corresponding 1st quartiles.

Next, we categorized the participants according to the presence of obesity, which was defined as a BMI ≥ 25 kg/m^2^ (Figure 3 and Table S2). After a full adjustment, the ORs and 95% CIs for NAFLD in the 4th quartile of the TyG-WC were 24.45 (16.51–36.19) for the non-obese population and 7.79 (5.86–10.35) for the obese population compared to the corresponding 1st quartiles. The adjusted ORs for the 4th HOMA-IR, TyG, and TyG-BMI groups were 5.52, 5.79, and 14.48 for the non-obese population and 4.32, 4.40, and 7.13 for the obese population, respectively. In general, the increase in the ORs according to the metabolic parameter quartiles was more prominent for the non-obese population than for the obese population (Figure 3 and Table S2).

### 3.3. ROC Curve of the HOMA-IR and TyG-RELATED Markers for the Identification of NAFLD

The metabolic parameters each showed a moderate prognostic performance for NAFLD. The highest AUC was demonstrated by the TyG-WC (AUC = 0.843), followed by the TyG-BMI (AUC = 0.837), TyG (AUC = 0.770), and the HOMA-IR (AUC = 0.758) (Table 2 and Figure S1). The TyG-WC had significantly higher AUC values than the other indices (*p* < 0.001 vs. HOMA-IR, *p* < 0.0001 vs. TyG, *p* = 0.014 vs. TyG-BMI). The AUC for each parameter was also higher for the non-obese population than for the obese population (AUC [HOMA-IR], 0.719 vs. 0.699; AUC [TyG], 0.755 vs. 0.698; AUC [TyG-BMI], 0.798 vs. 0.733; AUC [TyG-WC], 0.808 vs. 0.743, respectively [Table 2 and Figure S1]).

## 4. Discussion

In this study, we discovered that people with high TyG-related indices were more likely to have NAFLD, and these indices were more effective than HOMA-IR for detecting NAFLD. Among the different indices, the TyG-WC had the strongest association with NAFLD, as measured using ultrasonography. After controlling for confounding factors, the participants in the highest TyG-WC quartile were 40 times more likely to have NAFLD than those in the lowest TyG-WC quartile. According to the ROC analysis, the TyG-WC was the most reliable indicator for NAFLD among the parameters with a high discrimination power. Furthermore, we found that the discriminative value of the TyG-WC was higher for the non-obese patients in our subgroup analyses. Based on these findings, we propose that the TyG-WC is a useful marker for detecting patients with NAFLD, particularly in the non-obese population.

NAFLD is closely linked to IR in both the liver and adipose tissue [8,28,29,30] as well as to decreased whole-body insulin sensitivity [8,28,29]. Previous investigations have shown a decreased capacity of insulin to inhibit endogenous glucose synthesis in patients with NAFLD, indicating hepatic IR [28,29,30], as well as a 45–50% decrease in whole-body glucose disposal [28,29]. Furthermore, individuals with NAFLD exhibit defective insulin suppression of free fatty acids (FFAs), which is consistent with adipocyte IR [31,32,33,34]. These results suggest that IR is a major contributor to the pathophysiology of NAFLD, and a decrease in the insulin responsiveness at the adipocyte level may contribute to hepatic steatosis via an increased flow of FFAs to the liver [8]. Furthermore, a recent investigation including 143 patients with NAFLD revealed that WC was predictive for increased risk of fatty pancreas, which is associated with the endocrine and exocrine pancreas dysfunction [35].

Given its role in the pathophysiology of NAFLD, measuring IR may be helpful for identifying people who are at a high risk of developing NAFLD [36]. The gold standard for evaluating IR is the HEC [13]; however, this method is time consuming and unsuitable for practical use. HOMA-IR is now a widely recognized parameter for evaluating IR, and previous research has shown an independent link between NAFLD and the HOMA-IR [37,38]. However, measurement of insulin levels remains difficult to perform in many laboratories and clinics, and there are issues with standardization [39]. As a result, more accessible and useful laboratory markers for IR are required. In many investigations, the TyG index suggested by Guerrero–Romero et al. has shown excellent sensitivity and specificity in the diagnosis of IR; therefore, it may be used as an alternative index to evaluate IR [16]. Additionally, TyG has recently been linked to NAFLD; a cross-sectional study of 10,761 people in a Chinese health examination cohort showed that TyG was helpful for identifying individuals with NAFLD who had also been identified using ultrasonography [40]. Lee et al. [37] found that TyG performed better than the HOMA-IR in predicting NAFLD. Our results were similar to those of these two previous investigations, as we found that the TyG-index performed slightly better in identifying NAFLD than did the HOMA-IR (AUC 0.758 vs. 0.770).

Considering the role of obesity in IR and NAFLD, we hypothesized that the combination of the TyG index and obesity markers (i.e., TyG-BMI and TyG-WC) would perform better as indicators of NAFLD; this hypothesis was subsequently proven by our findings. Specifically, our results show that the discriminative ability of the TyG-WC for NAFLD was better than that of the other parameters with the highest ORs in the 2nd, 3rd, and 4th quartiles (Figure 3 and Table S2) and that the TyG-WC had the highest AUC (Table 2 and Figure S1). To date, there have only been a few investigations on the diagnostic effectiveness of the TyG-related indices for NAFLD [36,41,42]. In 2017, a cross-sectional study that was conducted by Zhang et al. [41] showed that the TyG-BMI was an effective marker for detecting NAFLD in a non-obese (BMI < 25.0 kg/m^2^) Chinese population; compared with the lowest quartile of the TyG-BMI, the multivariable-adjusted ORs were 2.4 (1.6–3.6), 6.4 (4.2–9.7), and 15.3 (9.8–23.9) for those in the 2nd, 3rd, and 4th quartiles, respectively [41]. Another Chinese study showed that an increase in the TyG-BMI in a normolipidemic and non-obese subset of the Chinese population was related to an increased incidence of NAFLD [36]. These results are similar to ours, as we found that an increased TyG-BMI was related to an increased incidence of NAFLD; however, the previous studies did not assess the diagnostic performance of the TyG-WC, and their study populations were limited to non-obese subjects.

In contrast, in a recent cross-sectional study of 184 overweight/obese adults from Iran, Khamseh et al. showed that there was a significant connection between TyG and its related parameters (i.e., TyG-BMI and TyG-WC) and the existence of NAFLD in overweight/obese people without diabetes [42]. In that study, the TyG-WC had the highest AUC for detecting NAFLD (0.693, 95% CI: 0.617–0.769), which is consistent with our findings (AUC of the TyG-WC in the obese population, 0.743, 95% CI: 0.728–0.759). However, their study only included obese persons, and the number of participants was rather small, which limited the generalizability of their findings [42]. Lee et al. reported that the TyG index and prevalence of NAFLD were significantly related, and the TyG index was superior to the HOMA-IR for predicting NAFLD in Korean adults; however, the authors did not assess the value of a combination of the TyG index and obesity indices (i.e., TyG-BMI and TyG-WC) [37]. To the best of our knowledge, the present study is the first to investigate the performance of TyG obesity indices in identifying NAFLD in a large cohort of healthy Korean individuals.

Our subgroup analyses revealed that the diagnostic performances of the TyG-related indices for NAFLD was good, especially for the non-obese patients. Although we cannot directly compare the diagnostic performances from different studies, the AUC of the TyG-BMI for the incidence of NAFLD was approximately 0.8 for the non-obese population (i.e., 0.835 [41], 0.8489 [36] and 0.798 in our subgroup analyses), while the values were lower in the obese population (0.675 [42] and 0.733 in our subgroup analyses). These findings imply that IR plays a critical role in the development of NAFLD in non-obese patients. Pre-existing metabolic risk factors may account for the majority of NAFLD development in obese adults, whereas non-obese people have those risk factors. As a result, the role of IR may be more pronounced in non-obese people; however, a more precise mechanism needs to be investigated further.

The following limitations were present in this investigation; first, as this was a cross-sectional observational study, the results cannot be assumed to indicate a causal relationship. Second, because our study included only Korean individuals from a single center, the results may be limited in their applicability to other ethnic groups. Third, because the current study compared TyG-related measurements to the HOMA-IR, rather than to the HEC technique, we were unable to offer data that supports the idea that the TyG-related indices are superior to HEC as the gold standard assessment of insulin sensitivity. Fourth, due to the retrospective nature of this study, the ultrasonographic diagnosis of NAFLD was based on one radiologist, so kappa coefficient to measure agreement between two radiologists could not be presented. Furthermore, there are several limitations of ultrasonography for the assessment of NAFLD. It is subjective and there is a lack of sonographic criteria for different degrees of steatosis [43]. In particular, the definitive diagnosis of NASH requires a liver biopsy [44,45], as ultrasonography cannot differentiate NASH from simple steatosis [46,47]. However, liver biopsy is invasive, and there is a possibility of procedure-related complications [43]. Ultimately, the high prevalence of NAFLD limits the use of liver biopsy as a routine method for risk assessment [43]. Recently, a systematic review and meta-analysis by Hernaez et al. [48] showed that ultrasound is a reliable diagnostic method for the detection of NAFLD when compared to histological diagnosis (AUC 0.93, a pooled sensitivity 84.8%, and a pooled specificity 93.6% for detecting ≥20–30% steatosis). This result suggested that ultrasonography could be a first-line imaging method in clinical practice and epidemiological research [49,50]. Finally, we were unable to collect a complete list of the medications that the participants were taking. As a result, because the participants taking herbal supplements or metformin for reasons other than diabetes were not screened at the time of the baseline health examination, we could not exclude those participants. Despite these limitations, this is the first study to examine the effectiveness of TyG-obesity-combined indices as a simple and cost-effective predictor of NAFLD across a large number of participants.

In conclusion, the findings from the current study reveal that the TyG-related indices were substantially associated with NAFLD. In detecting NAFLD in Korean adults, the TyG-related indicators outperformed the HOMA-IR. Among these indices, the TyG-WC was the most reliable marker for detecting NAFLD in healthy Koreans, especially in the non-obese population. The TyG-related indices can be easily calculated in clinics, as glucose, triglyceride, BMI and WC are routine measurements. Clinical implication of the TyG-related indices entails the screening of patients to be referred for ultrasonography and the selection of patients who need intensified lifestyle modification. In the field of medical research, these indices can be useful in detecting study subjects at greater risk of NAFLD for planning clinical trials or observational studies.

## Figures and Tables

**Table 1:** The baseline clinical and biochemical characteristics of the participants according to the presence of NAFLD.

	Total	No NAFLD	NAFLD	*p*
N (%)	10,585 (100)	7301 (69.0)	3284 (31.0)	<0.001
Age (years)	47.8 ± 8.7	47.3 ± 8.8	48.9 ± 8.3	<0.001
Sex (male, %)	6326 (59.8)	3692 (34.9)	2634 (24.9)	<0.001
Body mass index (kg/m^2^)	23.6 ± 2.8	22.7 ± 2.5	25.5 ± 2.5	<0.001
Waist circumference (cm)	80.3 ± 8.8	77.4 ± 7.9	86.8 ± 7.0	<0.001
Systolic BP (mmHg)	116.2 ± 14.1	114.1 ± 13.9	120.8 ± 13.6	<0.001
Diastolic BP (mmHg)	72.7 ± 9.0	71.3 ± 8.7	75.7 ± 8.9	<0.001
Current smoker (%)	4891 (46.2)	2855 (27.0)	2036 (19.2)	<0.001
Moderate drinker (%)	3869 (36.6)	2395 (22.6)	1474 (13.9)	<0.001
Physically active (%)	2302 (21.7)	1627 (15.4)	675 (6.4)	<0.001
Family history of diabetes (%)	2110 (19.9)	1385 (13.1)	725 (6.8)	<0.001
Hypertension (%)	1179 (11.1)	638 (6.0)	541 (5.1)	<0.001
FPG (mg/dL)	93.8 ± 9.2	92.4 ± 8.8	96.9 ± 9.4	<0.001
HbA1c (%)	5.3 ± 0.4	5.3 ± 0.4	5.5 ± 0.4	<0.001
HbA1c (mmol/mol)	34.9 ± 4.1	34.4 ± 4.0	36.2 ± 4.1	<0.001
Total cholesterol (mg/dL)	190.3 ± 32.0	186.7 ± 31.2	198.3 ± 32.3	<0.001
TG (mg/dL)	120.2 ± 73.8	101.2 ± 50.6	162.4 ± 96.5	<0.001
LDL-C (mg/dL)	122.0 ± 28.5	117.9 ± 27.6	130.9 ± 28.5	<0.001
HDL-C (mg/dL)	57.2 ± 14.1	60.1 ± 14.3	50.6 ± 11.1	<0.001
Uric acid (mg/dL)	5.2 ± 1.4	4.9 ± 1.3	5.9 ± 1.3	<0.001
AST (U/L)	22.2 ± 7.2	21.1 ± 6.4	24.7 ± 8.1	<0.001
ALT (U/L)	21.1 ± 11.7	17.9 ± 8.6	28.3 ± 14.2	<0.001
GGT (U/L)	24.0 ± 23.2	20.0 ± 19.6	32.7 ± 27.6	<0.001
hsCRP (mg/L)	0.1 ± 0.3	0.1 ± 0.2	0.2 ± 0.3	<0.001
HOMA-IR	1.5 ± 0.9	1.2 ± 0.7	2.0 ± 1.1	<0.001
TyG index	9.2 ± 0.5	9.0 ± 0.5	9.5 ± 0.5	<0.001
TyG-BMI	217.1 ± 33.2	205.2 ± 27.5	243.7 ± 29.1	<0.001
TyG-WC	740.4 ± 107.6	700.9 ± 91.9	828.2 ± 85.7	<0.001

BP, blood pressure; FPG, fasting plasma glucose; HbA1c, hemoglobin A1c; TG, triglyceride; LDL-C, low-density lipoprotein cholesterol; HDL-C, high-density lipoprotein cholesterol; AST, aspartate aminotransferase; ALT, alanine aminotransferase; GGT, gamma-glutamyl transferase; hsCRP, high-sensitivity C-reactive protein; HOMA-IR, homeostatic model assessment for insulin resistance; TyG, triglyceride-glucose; BMI, body mass index; WC, waist circumference; the *p*-value shows comparison between the no NAFLD and NAFLD groups.

**Table 2:** Area under the receiver operating characteristic curves for each parameter in the (**A**) total population, (**B**) non-obese population, and (**C**) obese population.

(A) Total Population.			
Parameter	AUC	Standard error	95% CI
HOMA-IR	0.758	0.005	0.750–0.766
TyG	0.770	0.005	0.762–0.778
TyG-BMI	0.837	0.004	0.830–0.844
TyG-WC	0.843	0.004	0.836–0.850
Pairwise comparison	Difference AUC	95% CI	*p*-value
TyG-WC vs. HOMA-IR	0.085	0.075–0.095	<0.001
TyG-WC vs. TyG	0.073	0.066–0.081	<0.001
TyG-WC vs. TyG-BMI	0.006	0.001–0.010	0.014
TyG-BMI vs. HOMA-IR	0.079	0.070–0.089	<0.001
TyG-BMI vs. TyG	0.067	0.059–0.076	<0.001
TyG vs. HOMA-IR	0.032	0.001–0.023	0.032
**(B) Non-Obese Population.**			
Parameter	AUC	Standard error	95% CI
HOMA-IR	0.719	0.007	0.708–0.729
TyG	0.755	0.007	0.745–0.764
TyG-BMI	0.798	0.006	0.788–0.807
TyG-WC	0.808	0.006	0.799–0.817
Pairwise comparison	Difference AUC	95% CI	*p*-value
TyG-WC vs. HOMA-IR	0.089	0.074–0.105	<0.001
TyG-WC vs. TyG	0.053	0.043–0.064	<0.001
TyG-WC vs. TyG-BMI	0.011	0.003–0.018	0.007
TyG-BMI vs. HOMA-IR	0.079	0.064–0.094	<0.001
TyG-BMI vs. TyG	0.043	0.033–0.053	<0.001
TyG vs. HOMA-IR	0.036	0.020–0.052	<0.001
**(C) Obese Population.**			
Parameter	AUC	Standard error	95% CI
HOMA-IR	0.699	0.010	0.682–0.715
TyG	0.698	0.010	0.681–0.714
TyG-BMI	0.733	0.009	0.717–0.749
TyG-WC	0.743	0.009	0.728–0.759
Pairwise comparison	Difference AUC	95% CI	*p*-value
TyG-WC vs. HOMA-IR	0.045	0.023–0.067	<0.001
TyG-WC vs. TyG	0.046	0.030–0.061	<0.001
TyG-WC vs. TyG-BMI	0.010	−0.003–0.024	0.130
TyG-BMI vs. HOMA-IR	0.035	0.014–0.056	0.001
TyG-BMI vs. TyG	0.035	0.021–0.050	<0.001
TyG vs. HOMA-IR	0.001	−0.022–0.023	0.952

The differences in the prediction performances between the parameters are presented as an ROC curve (AUC) between the models. AUC = area under the receiver operating characteristic (ROC) curves; CI = confidence interval.

## Supplementary Materials

The following are available online at https://www.mdpi.com/article/10.3390/jcm11010041/s1, Figure S1: Receiver operating characteristic (ROC) curve of the metabolic parameters for the identification of NAFLD in the (A) total population, (B) non-obese population and (C) obese population; Table S1: Proportion of the participants with NAFLD according to the HOMA-IR, TyG, TyG-BMI, and TyG-WC quartiles; Table S2: The ORs for NAFLD according to the parameters in (A) total population, (B) non-obese population and (C) obese population.
