# Supplementary material for: Triglyceride Glucose-Waist Circumference Is Superior to the Homeostasis Model Assessment of Insulin Resistance in Identifying Nonalcoholic Fatty Liver Disease in Healthy Subjects
Source: J Clin Med. 2021 Dec 23;11(1):41. doi: 10.3390/jcm11010041 (PMC8745545; doi:10.3390/jcm11010041)
Supplement: Supplementary file 1 [file jcm-11-00041-s001.zip › jcm-1471892-supplementary.pdf]

**Supplementary appendix**

**Figure S1.** Receiver operating characteristic (ROC) curve of metabolic parameters for identification of NAFLD in the (A) total population, (B) non-obese population and (C) obese population.

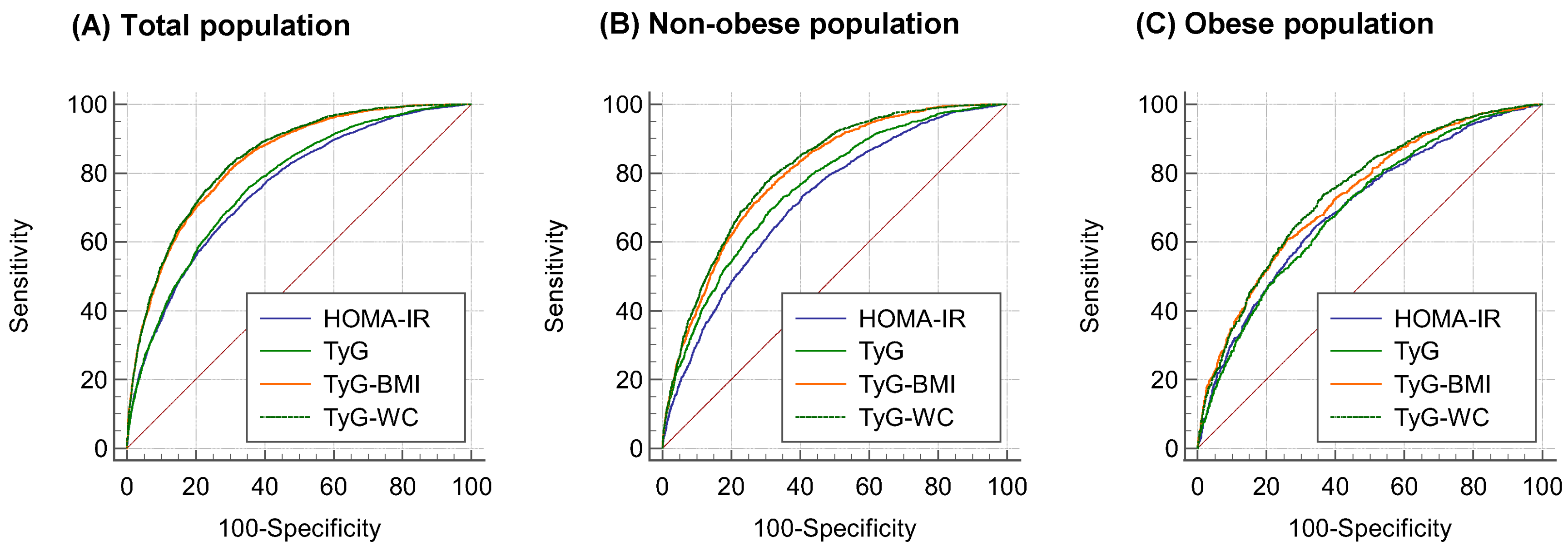

## Supplementary appendix

**Table S1.** Proportion of the participants with NAFLD according to the HOMA-IR, TyG, TyG-BMI, and TyG-WC quartiles.

| Parameter | Quartiles | | | |
|---|---|---|---|---|
| | Q1 | Q2 | Q3 | Q4 |
| HOMA-IR | | | | |
| NAFLD (-) | 2,404 (90.6) | 2,081 (78.9) | 1,743 (65.7) | 1,073 (40.6) |
| NAFLD (+) | 248 (9.4) | 557 (21.1) | 910 (34.3) | 1,569 (59.4) |
| TyG | | | | |
| NAFLD (-) | 2,439 (92.4) | 2,115 (57.9) | 1,677 (63.5) | 1,070 (40.4) |
| NAFLD (+) | 201 (7.6) | 539 (14.8) | 965 (36.5) | 1,579 (59.6) |
| TyG-BMI | | | | |
| NAFLD (-) | 2,555 (96.6) | 2,234 (84.3) | 1,686 (63.6) | 826 (31.2) |
| NAFLD (+) | 91 (3.4) | 413 (15.7) | 960 (36.4) | 1,820 (68.8) |
| TyG-WC | | | | |
| NAFLD (-) | 2,567 (97.0) | 2,246 (84.9) | 1,668 (63.0) | 820 (31.0) |
| NAFLD (+) | 79 (3.0) | 401 (15.1) | 978 (37.0) | 1,826 (69.0) |

Values are presented as number (%). P<0.001 for all data.

**Table S2.** The ORs for NAFLD according to the parameters in (A) total population, (B) non-obese population and (C) obese population.

(A) Total population

| Parameter | OR (95% CI) | | | |
|---|---|---|---|---|
| | Unadjusted | Model 1 | Model 2 | Model 3 |
| HOMA-IR | | | | |
| 1st quartile | reference | reference | reference | reference |
| 2nd quartile | 2.60 (2.21–3.05) | 2.49 (2.11–2.94) | 2.27 (1.92–2.68) | 2.09 (1.76–2.48) |
| 3rd quartile | 5.06 (4.34–5.90) | 4.78 (4.08–5.59) | 4.09 (3.49–4.81) | 3.45 (2.92–4.07) |
| 4th quartile | 14.17 (12.18–16.50) | 13.37 (11.43–15.64) | 10.43 (8.87–12.25) | 7.24 (6.12–8.56) |
| TyG | | | | |
| 1st quartile | reference | reference | reference | reference |
| 2nd quartile | 3.09 (2.60–3.67) | 2.60 (2.18–3.10) | 2.36 (1.97–2.82) | 2.09 (1.74–2.51) |
| 3rd quartile | 6.98 (5.93–8.23) | 5.30 (4.48–6.27) | 4.42 (3.72–5.26) | 3.76 (3.15–4.50) |
| 4th quartile | 17.91 (15.21–21.09) | 12.68 (10.73–15.00) | 9.51 (7.98–11.34) | 7.07 (5.89–8.50) |
| TyG-BMI | | | | |
| 1st quartile | reference | reference | Reference | reference |
| 2nd quartile | 5.19 (4.11–6.56) | 4.47 (3.53–5.65) | 4.09 (3.23–5.19) | 3.50 (2.75–4.45) |
| 3rd quartile | 15.99 (12.78–19.99) | 12.47 (9.93–15.67) | 10.85 (8.61–13.67) | 8.63 (6.83–10.92) |
| 4th quartile | 61.86 (49.42–77.45) | 46.56 (36.99–58.61) | 37.45 (29.59–47.41) | 25.34 (19.93–32.23) |
| TyG-WC | | | | |
| 1st quartile | reference | reference | reference | reference |
| 2nd quartile | 5.80 (4.53–7.43) | 6.08 (4.72–7.82) | 5.26 (4.08–6.78) | 4.72 (3.65–6.10) |
| 3rd quartile | 19.05 (15.03–24.16) | 21.02 (16.32–27.07) | 16.92 (13.09–21.86) | 13.28 (10.23–17.24) |
| 4th quartile | 72.36 (57.00–91.85) | 81.45 (62.82–105.62) | 62.18 (47.65–81.14) | 41.57 (31.66–54.59) |

Model 1 was adjusted for age and sex. Model 2 was adjusted for the variables included in model 1, plus alcohol, exercise, smoking, SBP, TC, hsCRP and HbA1c. Model 3 was adjusted for the variables included in model 2, plus AST, ALT and GGT

(B) Non obese population

| Parameter | OR (95% CI) | | | |
|---|---|---|---|---|
| | Unadjusted | Model 1 | Model 2 | Model 3 |
| HOMA-IR | | | | |
| 1st quartile | reference | reference | reference | reference |
| 2nd quartile | 2.23 (1.76–2.82) | 2.19 (1.72–2.77) | 1.99 (1.57–2.53) | 1.83 (1.43–2.35) |
| 3rd quartile | 4.23 (3.39–5.27) | 4.16 (3.33–5.21) | 3.68 (2.94–4.62) | 3.20 (2.54–4.04) |
| 4th quartile | 8.87 (7.18–10.97) | 8.93 (7.19–11.10) | 7.30 (5.85–9.11) | 5.52 (4.39–6.94) |
| TyG | | | | |
| 1st quartile | reference | reference | reference | reference |
| 2nd quartile | 2.56 (1.98–3.32) | 2.18 (1.68–2.83) | 1.98 (1.52–2.58) | 1.74 (1.33–2.28) |
| 3rd quartile | 5.24 (4.11–6.68) | 4.07 (3.18–5.20) | 3.43 (2.67–4.41) | 2.88 (2.23–3.72) |
| 4th quartile | 13.79 (10.91–17.42) | 9.86 (7.77–12.52) | 7.67 (5.98–9.83) | 5.79 (4.49–7.48) |
| TyG-BMI | | | | |
| 1st quartile | reference | reference | reference | reference |
| 2nd quartile | 4.38 (3.12–6.16) | 3.86 (2.74–5.43) | 3.56 (2.52–5.02) | 3.17 (2.24–4.48) |
| 3rd quartile | 11.14 (8.07–15.38) | 8.77 (6.33–12.15) | 7.60 (5.46–10.56) | 6.15 (4.41–8.59) |
| 4th quartile | 33.33 (24.30–45.70) | 24.21 (17.54–33.42) | 19.75 (14.22–27.42) | 14.48 (10.38–20.20) |
| TyG-WC | | | | |
| 1st quartile | reference | reference | reference | reference |
| 2nd quartile | 5.52 (3.78–8.06) | 5.49 (3.75–8.03) | 4.80 (3.27–7.04) | 4.38 (2.98–6.44) |
| 3rd quartile | 14.69 (10.23–21.10) | 14.83 (10.20–21.56) | 12.13 (8.31–17.69) | 9.89 (6.76–14.48) |
| 4th quartile | 44.93 (31.47–64.15) | 46.18 (31.57–67.56) | 34.88 (23.70–51.34) | 24.45 (16.51–36.19) |

Model 1 was adjusted for age and sex. Model 2 was adjusted for the variables included in model 1, plus alcohol, exercise, smoking, SBP, TC, hsCRP and HbA1c. Model 3 was adjusted for the variables included in model 2, plus AST, ALT and GGT

(C) Obese population

| Parameter | OR (95% CI) | | | |
|---|---|---|---|---|
| | Unadjusted | Model 1 | Model 2 | Model 3 |
| HOMA-IR | | | | |
| 1st quartile | reference | reference | reference | reference |
| 2nd quartile | 1.83 (1.49–2.24) | 1.80 (1.47–2.22) | 1.77 (1.43–2.18) | 1.67 (1.34–2.07) |
| 3rd quartile | 3.51 (2.84–4.34) | 3.42 (2.76–4.24) | 3.17 (2.55–3.95) | 2.70 (2.15–3.39) |
| 4th quartile | 6.43 (5.12–8.10) | 6.73 (5.32–8.51) | 5.82 (4.57–7.43) | 4.32 (3.36–5.57) |
| TyG | | | | |
| 1st quartile | reference | reference | reference | reference |
| 2nd quartile | 2.20 (1.80–2.70) | 2.08 (1.69–2.56) | 1.96 (1.58–2.42) | 1.91 (1.53–2.39) |
| 3rd quartile | 3.31 (2.69–4.09) | 3.07 (2.48–3.80) | 2.73 (2.19–3.42) | 2.56 (2.03–3.23) |
| 4th quartile | 6.61 (5.25–8.32) | 6.03 (4.77–7.62) | 5.09 (3.97–6.52) | 4.40 (3.39–5.70) |
| TyG-BMI | | | | |
| 1st quartile | reference | reference | reference | reference |
| 2nd quartile | 2.60 (2.11–3.19) | 2.41 (1.95–2.97) | 2.26 (1.83–2.81) | 2.11 (1.69–2.64) |
| 3rd quartile | 4.43 (3.57–5.49) | 4.13 (3.33–5.14) | 3.83 (3.06–4.80) | 3.41 (2.70–4.31) |
| 4th quartile | 11.02 (8.60–14.11) | 10.46 (8.14–13.43) | 9.15 (7.05–11.87) | 7.13 (5.44–9.34) |
| TyG-WC | | | | |
| 1st quartile | Reference | reference | reference | reference |
| 2nd quartile | 2.53 (2.05–3.11) | 2.61 (2.09–3.26) | 2.48 (1.97–3.11) | 2.31 (1.83–2.91) |
| 3rd quartile | 5.30 (4.26–6.59) | 5.51 (4.35–6.99) | 5.06 (3.96–6.48) | 4.32 (3.35–5.58) |
| 4th quartile | 11.26 (8.80–14.42) | 11.69 (8.98–15.21) | 10.19 (7.74–13.40) | 7.79 (5.86–10.35) |

Model 1 was adjusted for age and sex. Model 2 was adjusted for the variables included in model 1, plus alcohol, exercise, smoking, SBP, TC, hsCRP and HbA1c. Model 3 was adjusted for the variables included in model 2, plus AST, ALT and GGT
